# Focal Update on Immunotherapy and Liver Transplantation in the Era of Transplant Oncology

**DOI:** 10.3390/curroncol31090371

**Published:** 2024-08-28

**Authors:** Maen Abdelrahim, Abdullah Esmail, Taizo Hibi, Vincenzo Mazzaferro

**Affiliations:** 1 Section of GI Oncology, Houston Methodist Neal Cancer Center, Houston Methodist Hospital, Houston, TX 77030, USA; aesmail@houstonmethodist.org; 2 Weill Cornell Medical College, New York, NY 10065, USA; 3 Cockrell Center for Advanced Therapeutic Phase I Program, Houston Methodist Research Institute, Houston, TX 77030, USA; 4 Department of Pediatric Surgery and Transplantation, Kumamoto University Graduate School of Medical Sciences, Kumamoto 862-0976, Japan; 5 Department of Oncology, University of Milan, 20122 Milan, Italy; 6 Gastro-Intestinal Surgery and Liver Transplantation Unit, The Istituto Nazionale dei Tumori (National Cancer Institute) of Milan, 20133 Milan, Italy

**Keywords:** immunotherapy, liver transplantation, transplant oncology, ctDNA

## Abstract

Transplant oncology is an expanding area of cancer therapy that specifically emphasizes the use of liver transplantation (LT) as the preferred treatment for patients with manageable, but unresectable, tumors. The management and optimization of overall survival strategies, accompanied by an arguably decent quality of life, have been at the forefront of liver oncology treatment, as a plurality of all primary liver cancers are identified as either hepatocellular carcinoma (HCC) or cholangiocarcinoma (CCA), which are classified as highly aggressive malignancies and frequently remain asymptomatic until they progress to advanced stages, rendering curative procedures, such as resection, impractical. This has led to an increase in utilization of neoadjuvant interventions conducted prior to surgery, which has yielded favorable outcomes. Though this treatment modality has prompted further investigations into the efficacy of immune checkpoint inhibitors (ICPIs) as standalone treatments and in combination with locoregional treatments (LRTs) to bridge more patients into curative eligibility. This multidisciplinary methodology and treatment planning has seen multiple successful trials of immunotherapy regimes and combinate treatments, setting the groundwork for increasing eligibility through downstaging and “bridging” previously ineligible patients within stringent LT criteria. Surveillance after LT is a crucial component of transplant oncology. The emergence of circulating tumor DNA (ctDNA) has provided a novel approach to identifying the recurrence of cancer in its early stages. Recent research has focused on liquid biopsy, a technique that effectively identifies the dynamics of cancer. This is another innovation to demonstrate the rate at which transplant oncology is rapidly advancing, making the focus of care feel disorienting. Modalities of care are constantly evolving, but when a field is changing as rapidly as this one, it is imperative to reorient to the data and the needs of the patients. In this commentary, we reflect on the update’s utilization of ICPIs in neoadjuvant settings as well as the updates on the utilization of liquid biopsy in post-LT follow-up surveillance.

## HCC and CCA

The field of transplant oncology is an evolving area of cancer care and treatment with fascinating perspectives in clinical research, considering the outcome advantage of liver transplantation with respect to tumor resection in patients carrying limited tumor burdens. Hepatocellular carcinoma (HCC) and cholangiocarcinoma (CCA), the most prevalent hepatobiliary malignancies, collectively encompass over 90% of all liver cancer diagnoses. HCC and CCA are highly aggressive cancers that present virtually no symptoms until advanced stages of the disease have set in and a curative resection is a non-viable option [1,2,3,4]. Neoadjuvant studies before resection show promising results with better response rates, prompting ongoing trials examining immune checkpoint inhibitors (ICPIs) alone or combined with locoregional therapies (LRTs). Liver transplantation (LT) is the preferred treatment for unresectable HCC meeting accepted morphometric and biologic criteria or locally advanced disease partially downstaged with LRT, but the need for immunosuppression after LT conflicts with ICPIs’ immune augmenting effects, leading to caution in using them in this setting. Allocating the development of transplant oncology to multidisciplinary teams has shifted the goals of this treatment by adjusting drug regimens and surgical techniques in multiple trial studies focused on survival results and also through the expansion of the potentially curative option of LT to those patients in which ICPIs achieved consistent tumor response [5]. A focused aim of these multidisciplinary teams included scrutinizing patient selection criteria, which is essential for the improvement of future trials and the optimization of this modality of cancer care. Additionally, through the utilization of neoadjuvant therapies (Table 1), the population that may qualify for transplantation treatment has vastly expanded as treated patients fall within the boundaries of specific transplant criteria [6]. However, this increase in the number of LT-eligible patients directly exacerbates the greatest limitation of transplantation treatment, namely organ availability. In addition to the challenge of organ availability, follow-up care post-LT and the utilization of pre-LT immunotherapies and post-LT immunosuppressants are still a controversial topic when it comes to causative rejection and the nature of recurrence. The development of post-LT surveillance is a vital topic in transplant oncology. In this regard, circulating tumor DNA (ctDNA) is emerging as an additional modality of early recurrence surveillance under the purview of minimally invasive techniques.

The concept of downstaging is an important consideration in relation to LRTs. Systemic therapy and ICPIs have been increasingly used to “bridge” patients so that they may meet established eligibility criteria. Although the gold standard for LT is the Milan Criteria [13], there have been a multitude of expansions beyond the Milan Criteria. Close considerations of relevant research studies that explored viable transplant candidacy parameters have resulted in excellent outcomes. Neoadjuvant treatments have successfully been used to bridge established patient populations into LT (Table 1). Despite some previous results indicating a prevalence of adverse effects (AEs), published outcomes have shown that only 15% of patients with unresectable HCC had any AEs that required the discontinuation of treatment, concluding that ICPIs can be well tolerated by this patient population [14]. Several approved ICPI treatments have been studied in HCC populations to downstage LT criteria, and the results have shown a low rejection rate when a washout period is implemented prior to LT [12]. The recommended washout period after ICPI treatment varied between different reported studies [15]. Therefore, during the 2024 ILTS–ILCA Consensus Conference [16], the most contentious features of transplant oncology’s methodologies were evaluated and safe parameters were identified regarding downstaging criteria, macrovascular invasion, washout periods, recurrence care, immunotherapy setting utilization, and immunosuppressive interactions. The ILCA Consensus Conference’s recommendation for a washout period was 2–3 months, which allows the host system to readjust a hyperactive immune response before the LT procedure, thus lowering the risk of T-cell medication rejection. Adjustments in allocation policies to follow tumor response to ICPI and pre-LT washout, as well as future clinical trials, are needed for the establishment of more definitive recommendations [3,5,12]. ILTS–ILCA subcommittee members emphasized that clinical trial design in transplant oncology is complex, and it differs from known treatment strategies in oncology or transplant clinical research.

An increase in eligible cancer candidates for LT, though beneficial, calls for a heavier focus on patient selection for the organs available to be transplanted in order to minimize rejections. Though a feat in itself for the evolution of transplant oncology to downstage patients into the stringent criteria, such as Milan, there is a finite number of organs available for transplant utilization, and this severely limits the rate at which this field may expand [5]. Although living donor liver transplantation (LDLT) and dead donor liver transplantation (donor death declared from either brain death or following circulatory arrest—DBD and DCD, respectively) have recently had similar published results when it comes to recurrence and survival outcome data [17], this may be due to the LDLT recipient having to spend less time on the waitlist and, therefore, being less decompensated prior to LT. This topic remains highly controversial, especially since the health of both the donor and the recipient must be taken into consideration before LDLT surgical intervention. The development of patient selection criteria, by multidisciplinary teams—one of the most essential points for the future development of LT—must be based, however, on common ground, according to pre-determined thresholds for predicted overall survival and transplant benefits.

**Figure 1:**
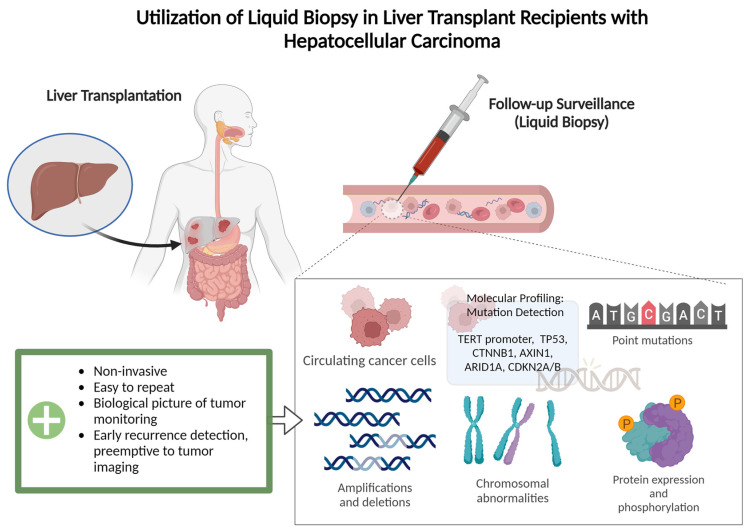
Illustrated process of post-transplantation surveillance by liquid biopsy. A non-invasive tool utilizing a patient’s blood sample for detection of minimal residual disease and an early detection of recurrence, which can be found prior to MRI/CT imaging.

With this in mind, preventing the recurrence of the disease is paramount to preserving the fragile state of global organ sharing today. Liver malignancies, HCC and CCA, have been shown to be highly recurrent diseases, so the utilization of accurate and non-invasive follow-up procedures post-LT is essential for the preservation of patients’ health [6,18,19]. Traditionally, post-LT surveillance consists of a strict regimen of scheduled imaging (CT/MRI) to evaluate for signs of metastases for years following treatment. The development of ctDNA surveillance, although with limited specificity in the pre-LT setting when the evaluation of response to various therapies is crucial, has shown promise in the identification of early recurrent disease after transplant [20,21] (Figure 1). A recent study described that the clearance of ctDNA was observed in 5 of 10 recipients who received sequential testing after LT for HCC, CCA, and colorectal liver metastases [22]. Used effectively, the ctDNA test can help eliminate the necessity for palliative treatment, and it can significantly improve the quality of life of this patient population [23,24]. However, comprehensive future studies are needed to explore the utility of ctDNA surveillance, especially in view of some study data that showed a spike in cell-free DNA following transarterial chemoembolization (TACE) [24,25,26].

The utilization of ICPIs in the neoadjuvant setting of transplant oncology has shown promising outcomes for patients. The recent focus of research on post-LT surveillance using liquid biopsy to assess ctDNA has potential as a malignancy-specific procedure to detect disease recurrence. All of these measures are continuously being advanced and studied to ensure efficacy and develop superior outcomes in transplant oncology. Ultimately, more rigorous investigators are encouraged to establish improved treatment options for this population in the transplant setting.

## Figures and Tables

**Table 1:** Utilization of ICPI in the pre-transplant setting of patients with HCC. Neoadjuvant intervention is primarily to “bridge” into designated transplant criteria. Abbreviations: M: male, F: female, UK: unknown, IST: immunosuppressive, ICPI: immune checkpoint inhibitor, HCC: hepatocellular carcinoma.

Age/Sex	ICPIs and Cycles	Timing Intervals btw ICPIs to LT	IST	Outcomes	Ref
14 M	Pembrolizumab 3	4.6 Months	Sirolimus Tacrolimus	No rejection	Kang et al. [7]
68 M	Nivolumab UK	10 Months	UK	No rejection	Peterson et al. [8]
60 M	Nivolumab 17	1.2 Months	Tacrolimus	No rejection	Dehghan et al. [9]
47 F	Nivolumab 1	4 Months	Tacrolimus Mycophenolate Steroid	Graft rejection	Chen, Z et al. [10]
39 M	Toripalimab 10 Lenvatinib UK	3.1 Months	Tacrolimus Methylprednisolone	Graft rejection	Chen, GH et al. [11]
64 M	Atezolizumab/Bevacizumab 6	4 months	Mycophenolate Tacrolimus	No rejection	Abdelrahim et al. [12]
61 M	Nivolumab 42	1 month	Mycophenolate Tacrolimus	No rejection	Abdelrahim et al. [12]
58 M	Lenvatinib 15 Atezolizumab/Bevacizumab 3	6 months	Mycophenolate Tacrolimus	No rejection	Abdelrahim et al. [12]
61 M	Nivolumab 1 Atezolizumab/Bevacizumab 3	2 months	Mycophenolate Tacrolimus Everolimus	No rejection	Abdelrahim et al. [12]
68 M	Atezolizumab/Bevacizumab 24 Lenvatinib 10	36 months	Mycophenolate Tacrolimus	No rejection	Abdelrahim et al. [12]
59 M	Nivolumab/Ipilimumab 1	41 months	Prednisone Tacrolimus Mycophenolate	No rejection	Abdelrahim et al. [12]
